# Surgical Treatment of Obesity. Special Mention to Roux-en-Y Gastric Bypass and Vertical Gastrectomy

**DOI:** 10.3389/fendo.2022.867838

**Published:** 2022-03-31

**Authors:** María José Luesma, José Fernando, Irene Cantarero, Pilar Lucea, Sonia Santander

**Affiliations:** ^1^ Department of Human Anatomy and Histology, School of Medicine, University of Zaragoza, Zaragoza, Spain; ^2^ General Surgery and Digestive System Service, Royo Villanova Hospital, Zaragoza, Spain; ^3^ Department of Morphological and Social Health Sciences, Faculty of Medicine and Nursing, University of Córdoba, Córdoba, Spain; ^4^ Department of Pharmacology and Physiology, School of Medicine, University of Zaragoza, Zaragoza, Spain

**Keywords:** obesity, bariatric surgery, gastric bypass, vertical gastrectomy, mixed techniques

## Abstract

**Introduction:**

The prevalence of obesity has increased exponentially in recent decades, being one of the diseases that most affects global health. It is a chronic disease associated with multiple comorbidities, which lead to a decrease in life expectancy and quality of life. It requires a multidisciplinary approach by a specialized medical team. Obesity can be treated with conservative or with surgical treatments that will depend on the characteristics of the patient.

**Objective/Methodology:**

The referenced surgery can be performed using different surgical techniques that are analyzed in the present work through an exhaustive narrative bibliographic review in the PubMed and Cochrane databases, as well as in UpToDate.

**Results:**

Currently, those most used are restrictive techniques, specifically vertical gastrectomy and mixed techniques, with gastric bypass being the “gold standard”.

**Conclusions:**

In order to choose one technique or another, the characteristics of each patient and the experience of the surgical team must be taken into account.

## Introduction

Obesity is a multifactorial chronic disease resulting from the interaction of environmental causes with the individual genotype, resulting from the excessive accumulation of body fat as a consequence of the imbalance between energy intake and expenditure. It is currently a pandemic in developed countries, a product of the change in lifestyle that is the second cause of preventable mortality after tobacco (1).

It is a disease with a complex interdisciplinary approach, responsible for multiple comorbidities (2). All patients in primary care should be screened by measuring weight, height, and BMI (Body Mass Index), this is the most used tool to quantify obesity and establish risk groups, although it has imprecisions, so it is advisable to also measure the abdominal girth, to differentiate between central or android obesity and peripheral or gynoid obesity (1, 3).

According to the World Health Organization (WHO) worldwide obesity has nearly tripled since 1975. In 2016, 39% of adults aged 18 years and over were overweight, and 13% were obese (4).

The estimated prevalence of obesity in Spain among those over 18 years of age is 21.6%. Obesity is more prevalent in men and increases with age. If we consider abdominal obesity, defined by waist circumference, the prevalence increases to 33.4% of the population, this being more frequent in women and also producing a progressive increase with age (3). The WHO considers overweight a BMI 25–29.9 kg/m^2^ and obesity a BMI greater than or equal to 30 kg/m^2^. In addition, the abdominal circumference is measured considering it pathological in men greater than 102 cm, and in women greater than 88 cm (5).

Obesity can be treated with conservative or with surgical treatments. There are degrees of obesity and also comorbidities [type 2 diabetes mellitus (T2DM), arterial hypertension, dyslipidemia, cardiovascular disease, sleep apnea–hypopnea syndrome and cancer, among others] in which it has been demonstrated that the benefit of conservative medical treatment is very limited compared to a surgical intervention, which constitutes the usual clinical practice. Bariatric surgery can be performed using different surgical techniques. Its analysis is the object of the present work.

## Methods

The study has been carried out through an exhaustive narrative bibliographic review, in the PubMed, Cochrane and UpToDate databases using the terms “obesity, bariatric surgery, gastric bypass, sleeve gastrectomy, intragastric balloon, endoscopic sleeve, endoluminal bypass, adjustable gastric band, mini gastric bypass, biliopancreatic diversion, gastroileal bypass, duodenal switch and single anastomosis duodeno-ileal bypass with sleeve gastrectomy”.

In the search process we included the following terms:

#1: “Obesity”[MeSH Terms] RESULT 231.874

#2: (((((((((((((((gastric bypass)) OR ((((gastrectomy[MeSH Terms])) AND ((sleeve))))) OR ((intragastric balloon))) OR (((endoscopic[MeSH Terms]) AND (sleeve)))) OR (((endoluminal) AND (bypass)))) OR ((adjustable gastric band))) OR ((mini gastric bypass))) OR ((one anastomosis gastric bypass))) OR ((biliopancreatic diversion))) OR ((gastro ileal bypass))) OR ((duodenal switch)))) OR ((single anastomosis duodeno-ileal bypass with sleeve gastrectomy)))) OR ((bariatric surger*[MeSH Terms])) RESULT 1715.

The search equation resulting from the combination of terms in PubMed was ((#1) and (#2)): #3. RESULT 1250.

Non-English- or Spanish-language articles, articles with very narrow areas of application, and articles without clear scientific evidence were avoided. Only primary surgical treatment has been considered in adult (19+ years).

## Treatment of Obesity

### Medical Treatment

Obesity, being a chronic disease with multiple associated comorbidities, requires individualized treatment, and the motivation of the patient to change habits is essential. The objective is to achieve weight loss and maintain it in the long term, improving comorbidities and avoiding future complications to obtain an improvement in quality and life expectancy.

Treatment of patients with BMI between 25 and 26.9 kg/m^2^ starts with a personalized eating plan, balanced hypocaloric Mediterranean diet combined with physical exercise, and minimum 150 min per week (30 min daily, 5 days/week). This treatment will be the basis for patients with a BMI greater than 27kg/m^2^ (2, 6, 7).

The goal is a 5–10% weight loss in 6 months, if the patient has a BMI greater than or equal to 35 kg/m^2^ you could pose a loss of 20%.

If these objectives are not met in patients with a BMI greater than or equal to 27 kg/m^2^ with comorbidities or BMI greater than or equal to 30 kg/m^2^ it is recommended to add pharmacological treatment. This should be suspended if after 3 months weight loss greater than 5% is not achieved (3).

The drug treatment options recommended by the Spanish and Portuguese Society for the Study of Obesity are liraglutide 3.0 (GLP-1 receptor agonist) as the first option. If it does not produce an effect, there is poor tolerance or it is contraindicated, it is replaced by orlistat 120 mg (gastric and pancreatic lipase inhibitor) or a combination of naltrexone 32 mg (opioid antagonist) with bupropion 360 mg (antidepressant dopamine reuptake inhibitor) and norepinephrine extended-release (8).

### Surgical Treatment

According to the 1991 consensus conference of the American National Institute of Health (NIH), candidates for bariatric surgery had to have a BMI greater than 40 kg/m^2^ or greater than 35 kg/m^2^ with associated comorbidities such as type 2 diabetes mellitus, arterial hypertension or sleep apnea–hypopnea syndrome (9). As these recommendations are nearly 30 years old, the American Society for Metabolic and Bariatric Surgery (ASMBS) recommended metabolic surgery should be offered as an option for suitable individuals with BMI 30–34.9 kg/m^2^ and obesity-related comorbidities (especially T2DM), who have not achieved substantial, durable weight loss and comorbidity improvement with reasonable nonsurgical methods (10, 11). The recommended age of the patient would be between 18 and 65 years. Outside of this range, it would be necessary to individualize each case. It is recommended that the patient has an absence of endocrine disorders, has the cognitive capacity to understand the treatment to adhere to the follow-up rules and, finally, not present psychiatric disorders, alcoholism or drug dependence (9, 12–14).

Preoperative weight loss of 5–10% is recommended as it presents intra/perioperative advantages, a shorter hospital stay, and greater adherence to life changes (1).

Its effectiveness is not without risks. The safety of bariatric surgery has been globally improved, assuming as standard a mortality of less than 0.5% and a morbidity of less than 0.7%. A technique that provides a good quality of life, has few side effects, and benefits more than 75% of patients is considered ideal. The choice of surgical technique depends on the goal of treatment, the individual assessment of cardiovascular risk, digestive or esophagogastric pathology, the profile of each patient and their preferences and, ultimately, the experience of the surgical team (2).

These techniques can be classified according to their mechanism of action as restrictive, malabsorptive and mixed (although at present malabsorptive techniques themselves no longer exist), and depending on the access route in endoscopic or surgical laparoscopic, which currently constitutes the route of choice. Some of them are in disuse, but they have been incorporated to have a global vision of the surgical approach.

The different techniques are discussed below.

#### Endoscopic Techniques

Even though they are not surgical techniques, they have been considered in this section since they are useful for the primary treatment of obesity in addition to being used as a treatment for surgical complications (1, 15). We distinguish:

##### Intragastric Balloon

It consists of the temporary placement of a balloon occupying the gastric lumen, generating a restriction of intake. It has not achieved comparable results with laparoscopic bariatric surgery (15).

##### Endoscopic Sleeve

Equivalent to vertical gastrectomy, it is indicated in patients who are not candidates for bariatric surgery according to their BMI or those who prefer not to undergo standard surgery. The stomach volume is reduced by 70%, using an endoscopic suture device of the greater curvature. The technique is effective as it achieves clinically significant weight loss. The rate of postoperative serious adverse events is low, around 2.2%. But since it is a recent technique, there have been no comparative studies with conservative treatment and very few studies comparing it with bariatric surgery (16, 17).

##### Endoluminal Bypass

It is a flexible tube-shaped lining that extends from the duodenum to the proximal jejunum, preventing food from passing to the intestinal villi in the first part of the small intestine. It is indicated mainly in patients with grade 1 and 2 obesity and T2DM due to its effect on glycemic homeostasis (1).

#### Restrictive Surgical Techniques

They are based on the reduction of the ingested volume. A small gastric reservoir is created with a narrow outlet. It generates a feeling of early satiety by slowing down the intake.

The two most notable are:

##### Adjustable Gastric Band

All candidates for bariatric surgery can be indicated, but the ideal prototype would be young women, with a BMI less than 50 kg/m^2^, who understand the technique and are predisposed to physical activity and changes in eating habits.

The approach is always laparoscopic using the “pars flaccid” technique that replaces the “perigastric” approach, but it has been proven that it produced a higher percentage of dilatations or slides (18, 19). It begins by sectioning the gastrophrenic ligament at the angle of this. The “pars flaccid” of the gastrohepatic ligament is then opened to reveal the base of the right diaphragmatic pillar. The fat is dissected from the gastroesophageal junction and a retrocardial angle is created towards the angle of His, where the band is inserted. The band closes and tunnels on the anterior face. The band is connected to a subcutaneous reservoir that allows the diameter to be readjusted through the injection of saline solution (1).

A 15-year follow-up study of adjustable gastric banding developed in 2013 (3,227 patients) showed no perioperative mortality for the primary placement or for any revisional procedures. There was a mean of 47.0% EWL (percentage of excess weight loss) (n = 714; 95% CI = 1.3) for all patients who were at or beyond 10-year follow-up. Revisional procedures were performed for proximal enlargement (26%), erosion (3.4%), and port and tubing problems (21%). The band was explanted in 5.6% (20). This study was completed in 2019 at a single center where 8,378 laparoscopic adjustable gastic band (LAGB) patients were followed for up to 20 years with an overall follow-up rate of 54%. No surgical deaths occurred. Weight loss at 20 years was 30.1 kg, 48.9%EWL and 22.2% total weight loss (%TWL). Reoperation rate was initially high but reduced markedly with improved band and surgical and aftercare techniques (21).

Its use has now decreased due to suboptimal weight loss in patients, associated mechanical complications, and the high rate of band reoperation, removal, or revision (22). The gastric band has been widely abandoned by surgeons in most countries but deserves its mention by patients who currently still have a gastric band and need management and possible complications.

##### Vertical Gastrectomy or Gastric Sleeve

The introduction of the laparoscopic vertical sleeve gastrectomy (LVSG) has seen a marked rise in usage, even overtaking Roux-en-Y Gastric Bypass (RYGB) (23, 24); in fact, it has become the most popular operation for the treatment of morbid obesity in the United States and worldwide due to its technical simplicity and palatability to patients (25, 26).

From a functional point of view, LVSG modulates the physiology by changes in gastric emptying. Various studies describe an increase in the rate of emptying after of it and seem to be linked to the starting distance of the section from the pylorus (27). LVSG also causes physical loss of principal producer of ghrelin, classically attributed to the fundus gastric (28). Being a purely restrictive procedure, the LVSG works to reduce the caloric intake of patients and decrease appetite through removal of ghrelin producing cells (25).

It is indicated in patients with morbid obesity (BMI greater than 40 kg/m^2^ or BMI greater than 35 kg/m^2^ associated with concurrent diseases). In patients with a BMI greater than 50 kg/m^2^ it can be used as the first stage of a surgery performed in two phases.

It may be the technique of choice in patients who require long-term oral pharmacological treatments whose absorption could be altered in the intestinal diversions, in patients with chronic intestinal diseases, with concomitant gastric pathology, hepatomegaly or cirrhosis, metabolic syndrome or with extreme ages (older 65 years or adolescents) (1).

The long-term data for outcomes from LVSG is still being developed (25). Even though data were insufficient for a meta-analysis of the sleeve gastrectomy developed in 2019, it showed that it generated a weighted mean of 57% EWL from the two small studies that were included in the systematic review (21).

Through this procedure, long-term weight loss and an improvement in concurrent diseases are achieved: T2DM, arterial hypertension, dyslipidemia, obstructive sleep apnea–hypopnea syndrome and arthralgias. Mainly through two mechanisms, decrease in gastric volume and anorectic action by decreasing the concentration of ghrelin when sectioning the gastric fundus (29–31).

Prognostic factors for weight loss after vertical gastrectomy have been identified: a preoperative BMI >50 kg/m^2^, the presence of comorbidities, age >50 years, and distance >5 cm from the pylorus negatively influence the results (32).

The surgical procedure begins by placing the patient in a semi-sitting position with the legs spread. The surgeon stands between the legs of the patient, with the first assistant on the right and if there is a second on the left. Although there may be variations, usually five trocars are placed. The optic trocar is placed in the midline, slightly displaced to the left. The second is located to the left and above the optic trocar, left paramedial, for the surgeon’s right hand. The third is the right paramedial, for the surgeon’s left hand. The fourth trocar in the right subcostal area for the Genzime-type atraumatic liver retractor or for the left-sided assistant and the fifth trocar, in the left upper quadrant for the helper on the right side (33–35).

Before starting the procedure, the liver retractor lifts the left liver lobe to expose the lesser omentum, gastric antrum, and pylorus. It is important to respect a distance of 4–6 cm from the antrum proximal to the pylorus, to respect the pyloric sphincter. The anterior face of the antrum is grasped and pulled upwards, thus raising the greater omentum and separating it from the transverse mesocolon. The horizontal greater curvature and the lower part of the vertical greater curvature are released, to continue releasing the upper part and the left pillar of the diaphragm. Once the posterior aspect is completely released, the gastric section can be continued. A 34-42 French gauge spark plug is placed up to the duodenum; thus, when cutting the laparoscopic stapler at its end, it contacts the spark plug. The section begins at the gastric antrum to the upper end of the stomach. To check the tightness of the staple, methylene blue is inserted through the nasogastric tube to tighten the gastric cuff. Finally, the gastrectomy piece is extracted into a bag (31).

Concerns with the LVSG in the long term revolve around development or worsening of gastroesophageal reflux disease (GERD) or weight regain. The evidence places the incidence *de novo* GERD between 0 and 34.9% (1, 25, 28). Another frequent surgical complication is gastric fistula, which is observed in 0.6–4.3% of cases. It occurs mainly in the gastroesophageal junction, the most feared being the one produced in the staple line. It is diagnosed especially in the first five postoperative days. In these cases, it is usually necessary to reoperate the patient (36). The risk of postoperative bleeding is less than 1%. Gastric stenosis is another complication, mainly at the level of the incisura angularis, but it is infrequent, occurring only in 0.7–4% of patients (37, 38). The main causes of mortality are lung embolism, cardiorespiratory failure, and complications of fistulas (39).

Weight loss and the benefits in comorbidities with this technique seem to be superior to the adjustable gastric band, and close to the results of malabsorptive techniques, but without presenting as many risks as these.

#### Mixed Surgical Techniques

They have a restrictive component, as in previous techniques, and a malabsorptive component, by reducing the intestinal absorption surface.

##### Roux-en-Y Gastric Bypass (RYGB)

Gastric bypass, or gastrointestinal bypass, is a mixed technique. It is restrictive due to the small gastric pouch that reduces food intake, and malabsorptive due to the bypass of the pancreatic duodenum that carries partially digested food to the distal intestine, leading to a malabsorption of sugars and fats, improving glycemic balance (40–42).

The mechanisms of action include mechanical restriction of ingested calories due to the small gastric pouch, mild malabsorption due to the bypassing of a reasonable portion of the jejunum, and hormonal changes ensuing from the two latter, like reduction of the production of ghrelin from the excluded gastric fundus, early secretion of peptide YY(PYY) from the distal ileum and changes in the levels of various incretins such as GLP1 (43).

RYGB is the most frequent procedure to treat the severely obese in Europe (44–46) particularly in the presence of gastroesophageal reflux or T2DM (44, 46, 47).

It is the reference technique for the severely and morbidly obese patients, producing 72% weight loss after two years. Its efficacy, with a remission of T2DM in 84% at two years, has made it evolve as one of the possible treatments for this pathology (metabolic surgery). On the other hand, concurrent diseases and cardiovascular risk decrease, in relation to the improvement in inflammation markers, endothelial dysfunction and atherosclerosis after bariatric surgery (40–42).

It is indicated for use in patients with stable or increasing obesity for five years or more, after failure of conservative treatment, with a BMI greater than or equal to 40 kg/m^2^ or greater than or equal to 35 kg/m^2^ with comorbidities. It is especially indicated for use in obese diabetics with metabolic syndrome and cardiovascular risk. It is the key technique in patients with a tendency to peck due to the malabsorptive effect and also in patients with gastroesophageal reflux as a second choice in the face of the failure of restrictive interventions (48).

The operation begins by placing the patient in a semi-sitting position. The surgeon will stand between the legs of the patient, with the first assistant on the right and if there is a second assistant on his left. Before the operation, a nasogastric tube, antibiotic prophylaxis, and compression stockings are placed on the lower extremities to prevent thromboembolic risk. Surgery begins by insufflating the pneumoperitoneum into the abdominal cavity. The five trocars are then placed in the supraumbilical region. The first one below the xiphoid appendix, slightly displaced to the left where the optic will go. The second and third are the working trocars, one at the right pararectal level and the other at the left pararectal level.

First, the liver is rejected with the retractor in order to gain access to the supramesocolic region. To expose the duodenojejunal angle, the free edge of the greater omentum is divided to the greater curvature of the stomach, and the transverse mesocolon is elevated.

To perform the gastric pouch, the lesser curvature begins by dissecting the lesser curvature to move towards the lesser sac. The nasogastric tube is removed and the first horizontal stapling is performed. This is continued with the vertical section parallel to the lesser curvature up to the angle of His. The proximal jejunal loop ascends to the gastric pouch. An enterotomy of the jejunal loop and a gastrotomy are performed in the declining part of the gastric stapling. The posterior wall is anastomosed with a linear stapler and then the anterior surface is sutured with resorbable thread. The biliary loop is divided, transforming the loop into an omega into a Y loop. The next step is to measure the alimentary loop from the gastrojejunal anastomosis, which should not be more than 200 cm long. On both handles, the alimentary and the biliary ones, an enterotomy is performed on the antimesenteric border of each one and they are anastomosed laterally with a linear stapler. Finally, the Petersen space (between the meso of the ascended alimentary loop and the transverse mesocolon) is closed with running sutures to avoid internal hernias. There are schools in which the loop foot is made first and then the gastric reservoir and gastrojejunal anastomosis are made (48).

Some complications may appear:

- Postoperative hemorrhages between 1 and 4%, frequently in the immediate postoperative period; as a consequence of bleeding from the anastomosis, staple lines, mesos section, or visceral injuries (49);
- Serious complications such as fistulas that can occur early, such as gastrojejunal anastomosis fistula, or late, gastrogastric fistula (50);
- Intestinal occlusion that can occur early, but is more frequent late. It occurs between 10 and 16% of cases normally due to an internal hernia. Currently its incidence has decreased due to the closure of mesenteric defects (51);
- Ulcers and strictures of the gastrojejunal anastomosis simultaneously or independently (52);
- Late-onset gallstones, since after weight loss after surgery, lithogenesis is favored. Therefore, prophylactic treatment will have to be given if there is no history of cholecystectomy (53);
- Dumping Syndrome (DS) with a prevalence between 15 and 70%, with a wide range of presentations. The symptoms usually occur within the first hour following a meal (early DS) and include vasomotor symptoms such as palpitations, profuse sweating, dizziness, flushing, hypotension, and gastrointestinal symptoms, namely, diarrhea, bloating, nausea, or abdominal pain. Late DS occurs one to three hours following a meal and is primarily characterized by hypoglycemia due to excess insulin secretion that leads to confusion, hunger, syncope, tremor, irritability, etc. (54). The gastro-jejunal (GJ) anastomosis required can be performed on the anterior or posterior gastric pouch wall. Anterior GJ is associated with lower prevalence of DS but more frequent weight regain (55);
- Nutritional Complications: low B12, low serum folate, Thiamine, Iron, Calcium, Vitamin D, Zinc, Copper, Selenium, Vitamin C deficiency (56);
- Inadequate weight loss or weight regain after (RYGBP) occurs in more than a quarter of patients for various reasons. Subjective reasons, like lack of discipline from the part of the patient or more objective reasons such as anatomical changes attenuating the mechanism of action of the procedure due to surgical complications such as the occurrence of a gastro-gastric fistula or a gastric pouch enlargement and even hormonal changes. Among the available remedying treatment options conversion of RYGBP to biliopancreatic diversion with duodenal switch (BPD-DS) seems to produce the best results with acceptable peri-operative morbidity (43).
- Mortality is due to systemic complications: thromboembolic, cardiac or respiratory, with the highest risk in patients over 55 years of age, men and with a BMI greater than 50 kg/m^2^ (57).

To overcome the technical limits of laparoscopy and to potentially flatten the learning curve, the robotic system was introduced into bariatric surgery. The robotic approach might improve the outcome in revisional bariatric surgery. Its high cost is a major handicap, so its role in bariatric surgery is still unclear (46, 58).

##### Mini Gastric Bypass or One Gastic Bypass Anastomosis (MGB-OAGBP)

One anastomosis gastric bypass has gained popularity in recent years. It is now the third most commonly performed bariatric surgery worldwide after sleeve gastrectomy (SG) and Roux-en-Y gastric bypass (RYGB) (44). Its advantages include short operative time, a single anastomosis, acceptable rates of short-term complications, and effective weight loss (59).

Given that the Y-shunt is currently the procedure of choice, the OAGBP presents less technical difficulty than the Y-shunt due to the need for a single anastomosis (omega shunt) between the gastric tube and the jejunal loop. It achieves weight loss and resolution of T2DM comparable to gastric bypass (60).

Although this technique is gaining popularity, surgeons are reluctant to perform it because the long-term results are not well defined and prospective data are not available. In addition, there is controversy regarding its safety because omega mounting exposes them to have a greater probability of bile reflux in the gastric bag, and ulcers. In the studies carried out, it has also been seen that the omega shunt has a higher risk of protein and lipid malabsorption compared to the Y shunt, producing cases of severe malnutrition (61, 62).

In a systematic review of 12,807 MGB-OAGBP procedures described that the overall mortality was 0.10% and the leak rate was 0.96%. The follow-up duration ranged from 6 months to 12 years. A marginal ulceration rate of 2.7% and an anemia rate of 7.0% were reported. Approximately 2.0% of patients reported postoperative gastro-oesophageal reflux and 0.71% developed malnutrition. Excess weight loss at 6, 12, 24, and 60 months was 60.68, 72.56, 78.2, and 76.6% respectively. T2DM and hypertension resolved in 83.7 and 66.94% respectively. It concluded that there is now sufficient evidence to include MGB-OAGB as a mainstream bariatric procedure (63).

Therefore, it is not part of the recommended reference techniques, although in cases of massive abdominal obesity with thick and short mesos that limit the performance of the Y-shunt, it could be indicated (48).

##### Biliopancreatic Diversion

A common loop of 100–150 cm is made from the ileocecal valve and a posterior gastric section without gastrectomy, and gastrojejunal anastomosis.

It is indicated for use in patients with very high BMI, even above 60 kg/m^2^, since it is the intervention with the greatest malabsorption, also in patients with T2DM and other comorbidities since it favors its resolution, and in those patients who do not want to change their eating habits, since the food restriction is decreasing in time, being able to eat normally three months after the intervention (1).

##### Gastroileal Bypass

Gastroileal bypass is a modification of the biliopancreatic diversion technique. To avoid postoperative complications, it is performed without gastrectomy. It can be executed in two stages in patients with cardiac and respiratory risk. First, the gastroileal anastomosis is performed and secondly, the biliary intestine is divided by anastomosing it 100 cm from the ileocecal valve. The first stage of this intervention is what is currently called gastroileal bypass.

It is a simple, fast, safe and reproducible technique, obtaining excellent results. It is minimally restrictive, with great malabsorption. It can be used in patients with a BMI greater than 35 kg/m^2^ or with T2DM and BMI between 30 and 35 kg/m^2^ (64).

##### Duodenal Switch

An alternative to the previous technique, a vertical gastrectomy is performed with preservation of the pylorus and the duodenum is divided. At 300 cm from the ileocecal valve, the mesentery is divided into the duodenal loop and the biliopancreatic loop. The duodenal loop ascends retrocolically through the mesenteric orifice and anastomoses with the gastric pouch (duodenoileal anastomosis). Subsequently, an anastomosis is made between the biliopancreatic loop and the common loop 100 cm from the ileocecal valve. It is the preferred choice in patients with a BMI greater than 50 kg/m^2^ and with comorbidities, since it has been shown to have the best weight loss results and their resolution. Additionally, it has the benefit of being able to eat all kinds of food, improving the quality of the intake (1, 65).

##### Single Anastomosis Duodenoileal Bypass With Sleeve Gastrectomy (SADI-S)

It is an alternative to the duodenal switch. It consists of a single duodenoileal anastomosis together with a vertical gastrectomy. The duodenum is anastomosed directly to the omega loop of the ileum 200 cm from the ileocecal valve. The main benefit is a reduction in operative risk by performing only one anastomosis.

It is worth mentioning that the biliopancreatic diversion, the cost bypass, the duodenal switch and the SADI-S conceptually belong to the same type of interventions with some distinction.

SADI-S has been demonstrated to be a safe and reproducible technique and offers good weight loss results in the short term (66, 67). Results on diabetes are comparable to those obtained with the duodenal switch (68), and some studies have even found a better metabolic effect thanks to the longer common channel (69, 70).

There is no medical evidence to demonstrate the superiority of the duodenal switch over SADI-S, or about the long-term medical safety or efficacy of SADI-S (71). A study has recently been published reporting the 5 to 10-year outcome of a series of 164 patients consecutively submitted to primary SADI-S. There was no mortality. One patient had a gastric leak, and 2 patients had an anastomotic leak. A total of 25% of the patients were lost to follow-up at 10 years. Excess weight loss and total weight loss were 87 and 38% at 5 years and 80 and 34% at 10 years. A total of 12 patients were submitted to revisional surgery for hypoproteinemia. Preoperatively 41 diabetics were under insulin treatment; at 5 years, 7 remained with insulin and 12 at 10 years. Mean glycemia was 104 mg/dl at 5 years and 118 mg/dl at 10 years. Mean HbA1c was 5.51% at 5 years and 5.86% at 10 years. This allows concluding that in the long term, SADI-S seems to offer satisfactory weight loss and comorbidities resolution but there is insufficient data to comment on the long-term safety and efficacy of SADI-S (70). However, more studies should be done.

## Discussion

Bariatric surgery is a very effective treatment in the control of obesity, not only in terms of effective and sustained weight loss over time, but also in the resolution or improvement of associated comorbidities and in the improvement of quality of life (72).

Within surgical techniques, the Roux-en-Y gastric bypass is considered the gold standard; however, vertical gastrectomy is being performed with increasing frequency despite the lack of evidence of its long-term efficacy. That is why we are going to focus on them within all the exposed techniques.

**Figure 1:**
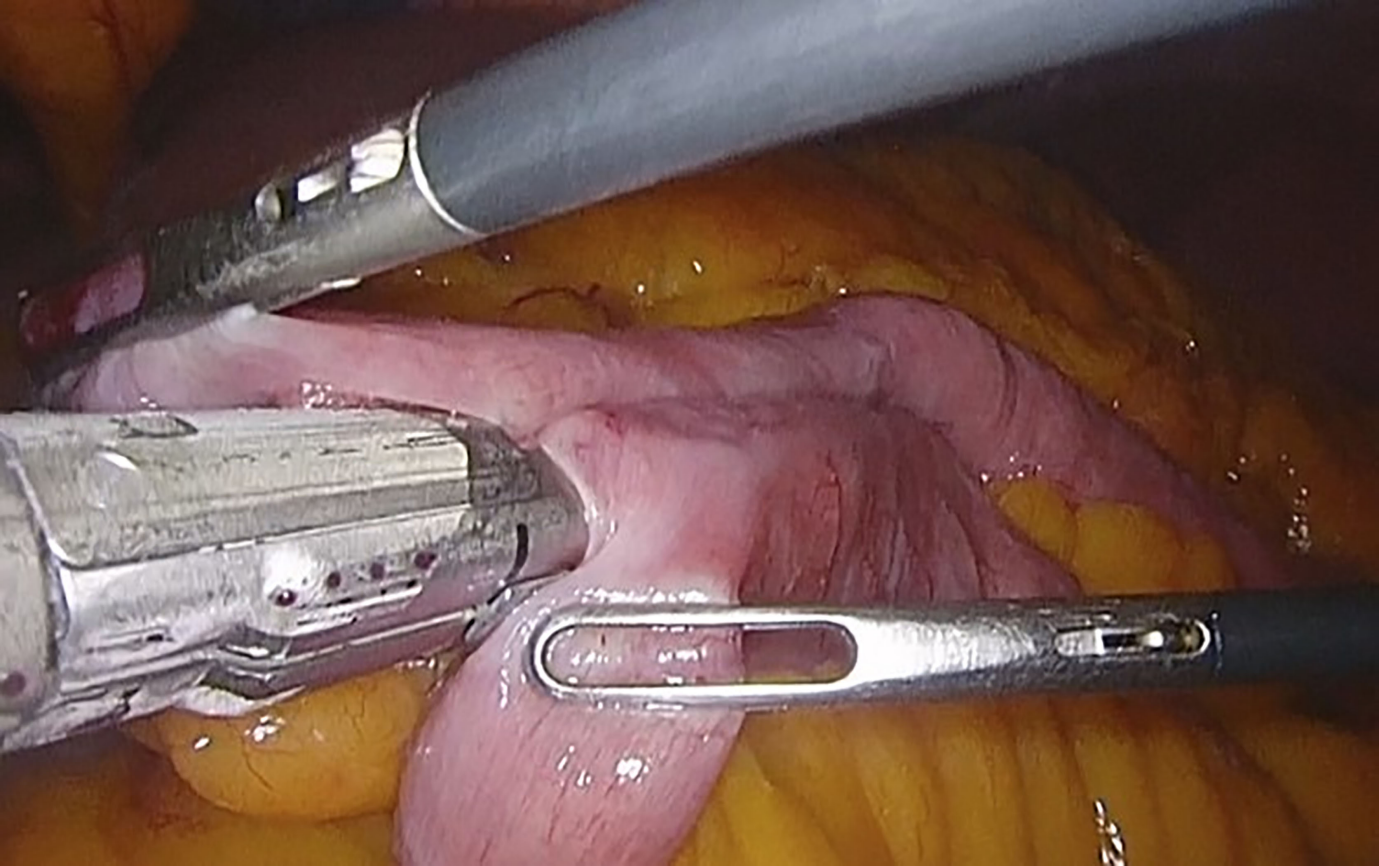
Jejunojejunal anastomosis in the loop foot with a mechanical endostapler. Taken from the General Surgery and Digestive System Service, Hospital Royo Villanova.

**Figure 2:**
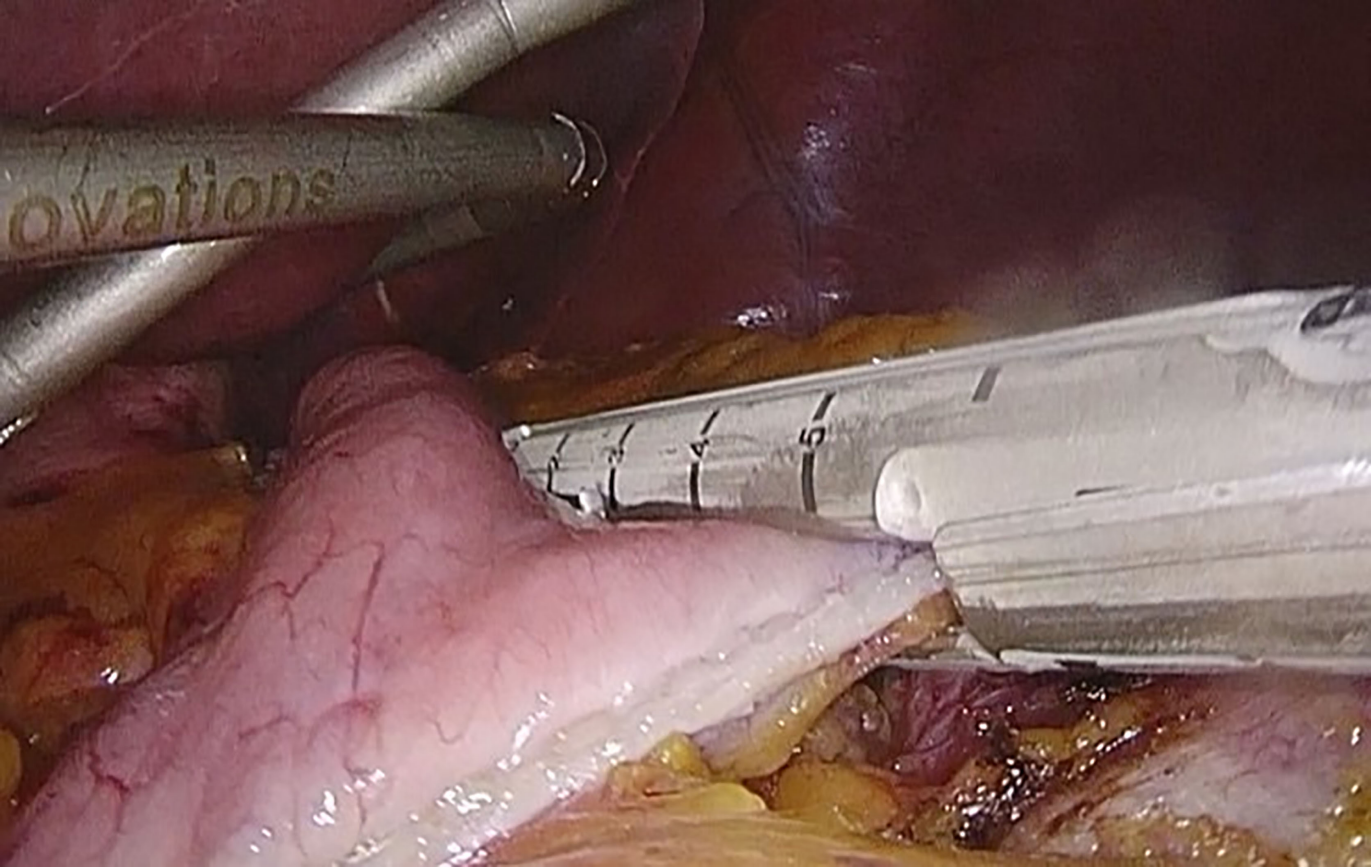
Linear mechanical stapling of the stomach, to make the gastric reservoir. Taken from the General Surgery and Digestive System Service, Hospital Royo Villanova.

**Figure 3:**
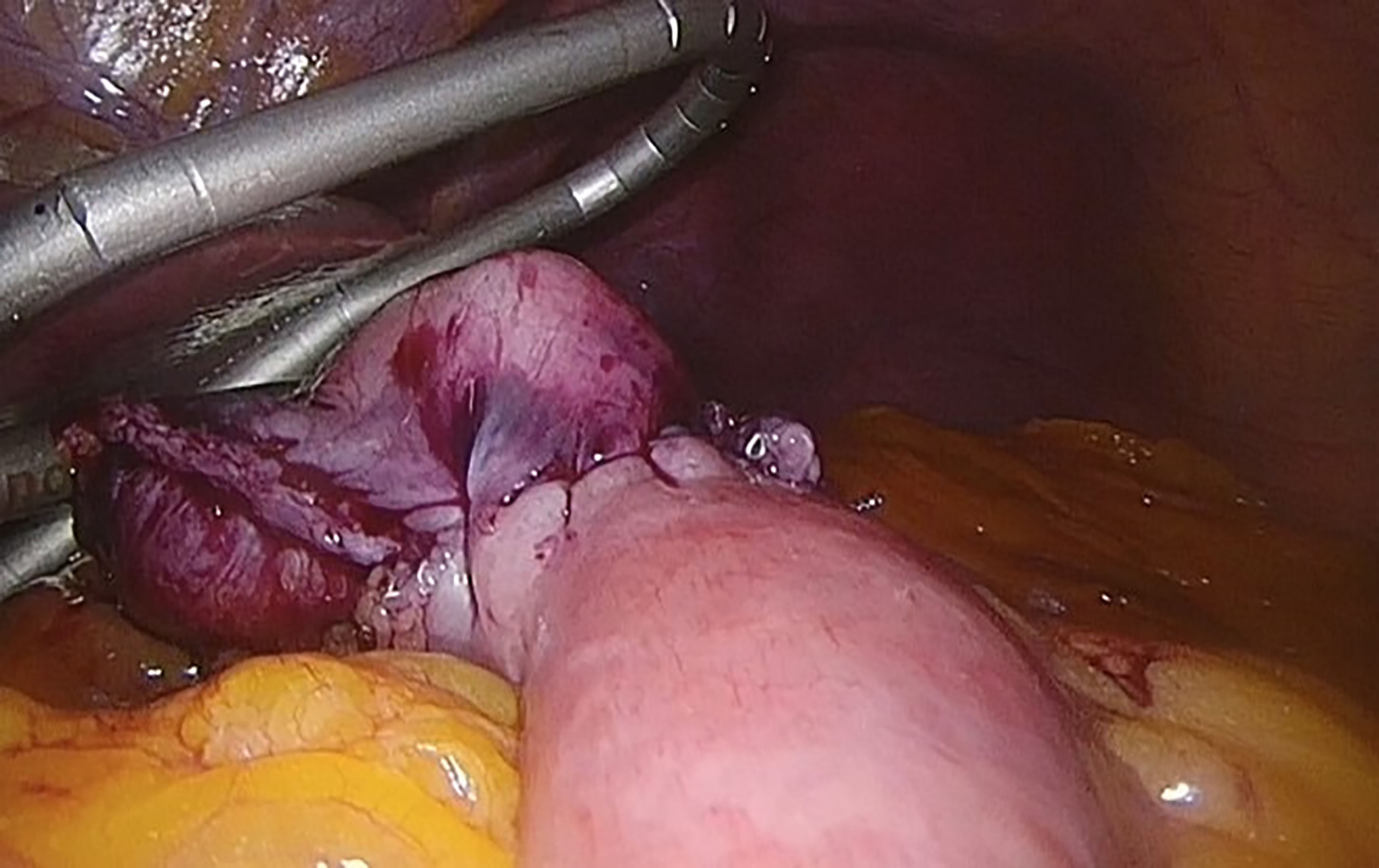
Gastrojejunal anastomosis. Taken from the General Surgery and Digestive System Service, Hospital Royo Villanova.

In the vertical gastrectomy procedure, most of the gastric curvature is removed and the stomach is tubulized (73, 74). In the Roux-en-Y procedure, the restrictive part of it is produced thanks to the small gastric reservoir that is created together with the division of the jejunum and anastomosing the distal part of it to the gastric reservoir, generating the food loop through which intestinal transit passes; and, on the other hand, the malabsorptive part of the technique, starting from the proximal end of the sectioned jejunum, anastomoses to the alimentary loop. This is how the biliopancreatic loop is formed where the biliary and pancreatic digestive secretions flow (**Figures 1**–**3**).

From the anastomosis where the two loops converge, the common loop is constituted where secretions and enzymes join with food products. The more distal the junction between the alimentary loop and the biliopancreatica, the greater the malabsorptive component and therefore the greater its effect on weight and the greater the probability of presenting nutritional and digestive complications (75).

**Figure 4:**
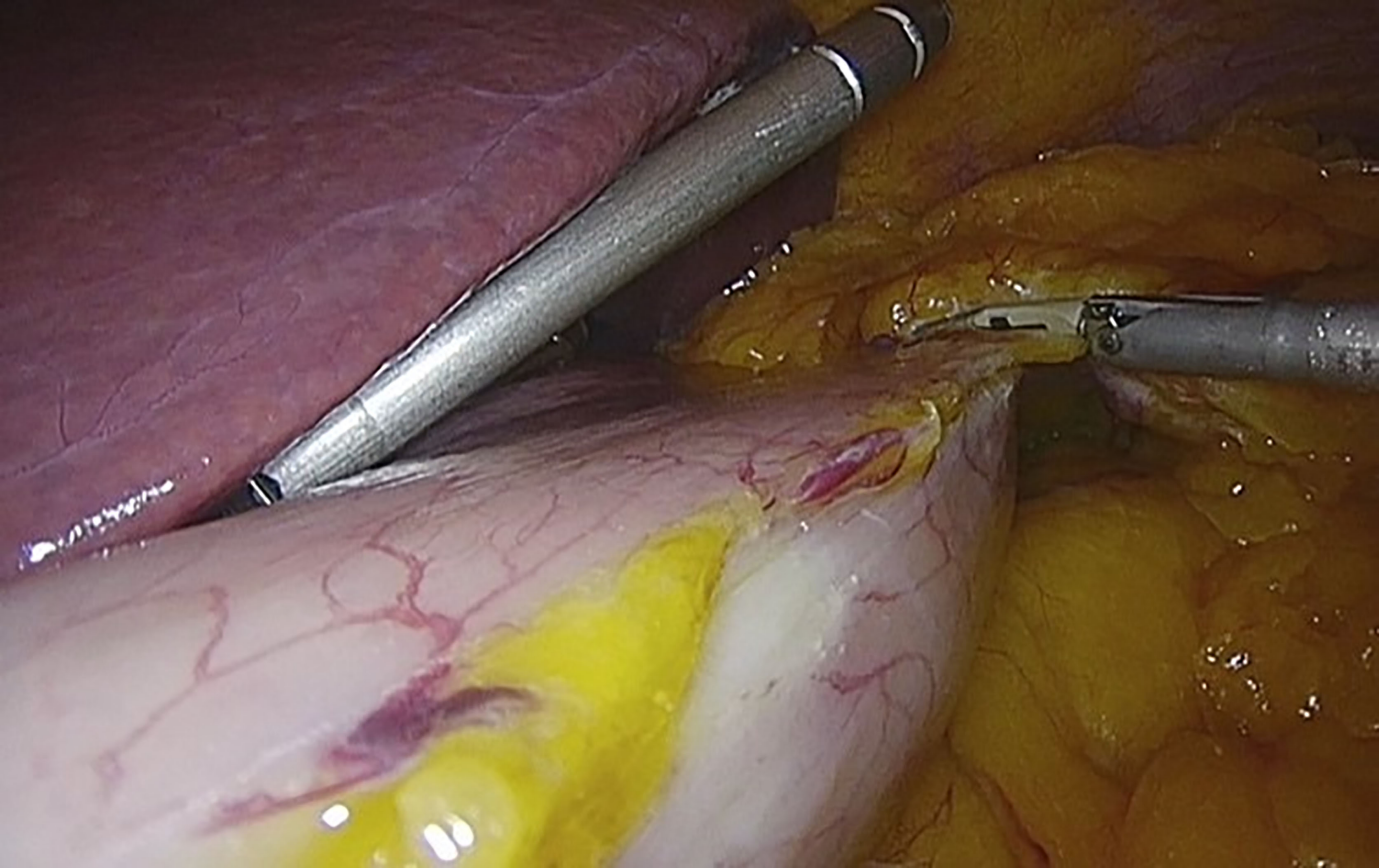
Release of the horizontal major bend and the bottom of the vertical major bend using the Thunderbeat. Taken from the General Surgery and Digestive System Service, Hospital Royo Villanova.

**Figure 5:**
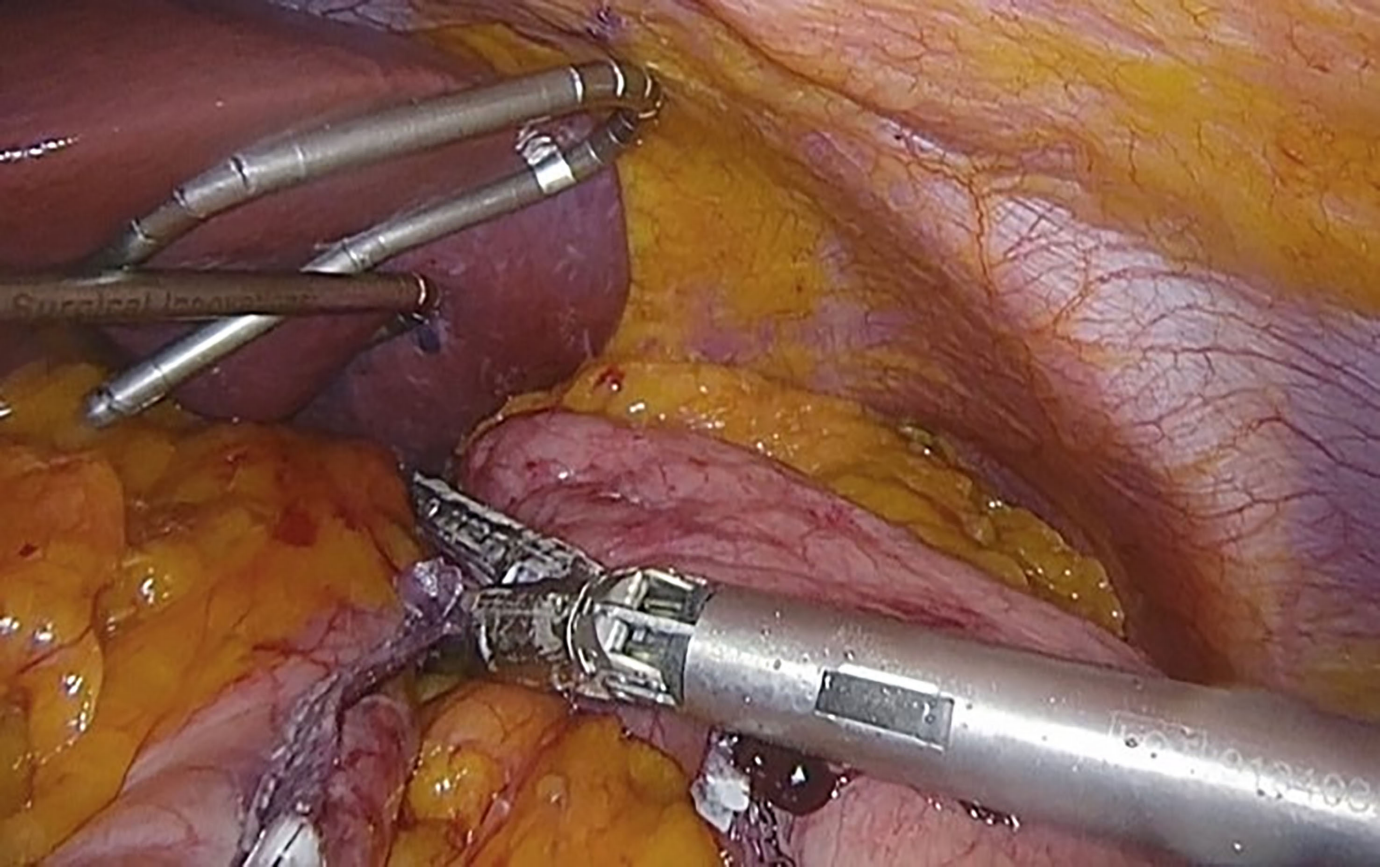
Stapling and sectioning of the gastric curvature using the mechanical endostapler. Taken from the General Surgery and Digestive System Service, Hospital Royo Villanova.

In any case, gastrectomy compared to bypass is technically easier as it does not require multiple anastomoses, it is faster and potentially safe, and the risk of internal hernias and protein and mineral malabsorption is reduced (73, 74) (**Figures 4**, **5**).

But in order to make an adequate comparison between both procedures, it is necessary to take into account effectiveness criteria such as changes in weight, comorbidities and long-term quality of life, to evaluate safety through the complications that may arise. To assess weight loss, it is recommended to use the percentage of excess BMI lost, which must be greater than 50% one year after the intervention and the percentage of total weight lost at 2 and 5 years (12).

The percentage of excess weight lost obtained by bypass is 70% in two years and between 50 and 60% at 5 years. Vertical gastrectomy achieves results clinically not inferior to bypass. In relation to the percentage of excess BMI lost at 5 postoperative years, they are 68.3% in vertical gastrectomy and 76% in gastric bypass (34, 76).

The systematic review of weight loss at 10 or more years for bariatric procedures developed by O’Brien et al. collected eighteen reports of gastric bypass that showed a weighted mean of 56.7%EWL and 2 reports of sleeve gastrectomy showed 58.3%EWL. The meta-analyses of eligible studies demonstrated comparable results (21). Only one study has reported longer follow-up to 25 years (198 patients). For transected RYGB patients, it reported 29.9% EWL at 20 years (N = 53). They reported a net weight gain at 25 years for their gastroplasty patients (21, 77). Long-term data on sleeve gastrectomy are modest at this time (21).

Regarding associated comorbidities such as: hypertension, sleep apnea syndrome, arthralgia, depression and hyperuricemia, which do not show statistically significant changes in favor of one or the other procedure 3 or 5 years after surgery. The remission rates of comorbidities show the same effects except in dyslipidemia and hypertension, observing that the total loss of cholesterol and LDL is significantly higher after 5 years postoperatively in the bypass. Regarding gastroesophageal reflux, improvement is greater in bypass, since symptoms worsen more frequently or develop *de novo* symptoms in vertical gastrectomy (78–80). LRYGB may be beneficial to gastroesophageal reflux disease (GERD) improvement but LSG may worsen GERD symptoms and may lead to *de novo* GERD (80).

The mortality rate for both surgeries is below 0.5% (0.40% for bypass and 0.36% for gastrectomy). If it is taken into account that the mortality of non-operated morbidly obese patients is higher than 6%, the efficacy of the techniques is verified (72).

The differences in the improvement of glycemic control in patients with T2DM between the two procedures are still controversial. The STAMPEDE trial (comparing medical treatment with bariatric surgery in T2DM over a 5-year period) is the largest to date. Regarding glycosylated hemoglobin (HbA1c) less than 6%, both techniques are superior to medical treatment, but no statistically significant differences were found between the two groups, but taking into account other criteria such as the number of drugs used or dependence on the insulin showed the superiority of Roux-en-Y gastric bypass over gastrectomy (81, 82). A recent review (2020), where it synthesized the best available evidence comparing LVSG with LRYGB for management of T2DM, reaches the same conclusions. It reports that both procedures are very effective at improving T2DM care, especially compared with conventional medical management. However, there may be a modest benefit to be had by using LRYGB over LVSG (24). Finally, a claims-based cohort study conducted in 2021 favored the continued use of RYGB over VSG among bariatric candidates for whom diabetes medication discontinuation is of paramount importance. However, the magnitude of the differences between RYGB and VSG was clinically small and the long-term durability of changes is unknown. Thus, it will be important for patients to consider other outcomes, such as long-term safety and side effect profile, when choosing a procedure (26). It is therefore necessary to carry out more studies before the longer-term results for durability of diabetes outcomes can be elucidated.

Base on this, when choosing the type of operation, the main determinant should be the co-morbidities such as dyslipidemia, hypertension and GERD of patients, not the BMI or T2DM (80).

In relation to patients with cardiovascular risk factors, bypass could be a better option since there has been an improvement in markers of inflammation, endothelial dysfunction, and atherosclerosis after the same (40, 78).

The percentage of major complications is slightly higher for bypass (2.5–3.6%) compared to vertical gastrectomy (2.2–2.4%), but no significant differences have been found in complications that require revision surgical or endoscopic the first 5 postoperative years. Although the complications they present are different, their frequency is not. In bypass, the most frequent causes of revision are internal hernia (but currently with the closure of mesenteric defects the incidence is reduced), rapid gastric evacuation syndrome or small bowel obstruction. On the other hand, in vertical gastrectomy they are due to gastroesophageal reflux, and insufficient weight loss (72, 79, 83).

Regarding short term outcomes, an analysis of the NSQIP database analyzed 24,117 patients who underwent LVSG or bypass for morbid obesity. When compared with RYGB patients who had a LVSG had a shorter operative time (101 vs. 130 min), and lower rates of blood loss requiring transfusion (0.6% vs. 1.5%), deep wound infection (0.06% vs. 0.2%), serious morbidity rate (3.8% vs. 5.8%), and 30-day reoperation rate (1.6% vs. 2.5%) (24, 84). An analysis of the 2015 MBSAQIP database of 134,142 patients demonstrated a lower mortality rate (0.1% vs. 0.2%), morbidity rate (5.8% vs. 11.7%), and leak rate (0.8% vs. 1.6%) in patients undergoing LVSG when compared to RYGB (24, 85). On the other hand, a direct comparison to the LRYGB was performed by randomizing patients to undergo RYGB or LVSG. The SLEEVEPASS trial enrolled 240 patients to undergo LVSG or RYGB. At six months, patients in the sleeve group when compared to patients in the bypass group had similar rates of excess weight loss (49.2% vs. 52.9%), resolution or improvement in diabetes (84.3% vs. 93.3%), hypertension (76.8% vs. 81.9%), and hypercholesterolemia (64.1% vs. 69.0%) (24, 86).

Regarding long term outcomes, the same SLEEVEPASS trial, five-year data of that trial reported mean excess weight loss of 49% in sleeve patients compared to 57% in bypass patients, a difference which was not statistically significant. The SM-BOSS trial of 217 Swiss patients randomized to undergo LVSG or RYGB demonstrated similar weight loss outcomes between the two groups, and similar rates of resolution of diabetes and hyperlipidemia (24, 79) but we must not forget, as has been said before, that long-term data for outcomes from LVSG is still being developed.

A recent study shows that, regardless of the technique used, the reduction in body weight contributed to the increase in serum concentrations of vitamin D. However, the sleeve surgical technique seems to significantly contribute to increase the serum concentrations of this nutrient after the surgery, possibly due to the lower disabsorptive power of this surgical technique (87).

A relevant factor when performing any surgical intervention is the improvement in the quality of life of patients. In this case, both procedures increase the quality of life at one year and at 5 postoperative years according to the gastrointestinal quality of life index and the BAROS quality of life index (78, 79).

So far there is no clear evidence to show that one of these two procedures is superior to the other in terms of long-term outcome (11, 12, 88) (**Table 1**).

**Table 1:** Obesity: Gastric bypass versus vertical gastrectomy.

**WEIGHT** % Excess Weight Loss (% EWL) 70% bypass in two years—vertical gastrectomy NOT inferior results % Excess BMI Lost (% EBMIL) at 5 years 76% bypass—68.3% vertical gastrectomy **COMORBIDITIES** No significant changes between the two techniques in: hypertension, sleep apnea syndrome, arthralgia, depression and hyperuricemia Exception: Dyslipidemia (total cholesterol, LDL) and gastroesophageal reflux is superior to the bypass **MORTALITY** 0.40% bypass—0.36% vertical gastrectomy **DIABETES MELLITUS 2** Both techniques are superior to medical treatment, without significant differences in glycosylated hemoglobin less than 6% Regarding the number of medications or insulin dependence, the bypass is higher **CARDIOVASCULAR RISK FACTORS** Bypass is a better option: it improves inflammation markers, endothelial dysfunction and atherosclerosis **COMPLICATIONS** Different complications but their frequency is not (2.5%) **QUALITY OF LIFE** Both procedures increase quality of life

Therefore, when choosing the technique, the fundamental objective of the treatment must be taken into account, the individual assessment of cardiovascular risk and digestive pathology, preferences and profile of the patient and, last but not least, the experience of the surgical team, since both bypass and vertical gastrectomy offer similar results in terms of quality of life and both produce better results than medical treatment (12).

## Conclusions

Bariatric surgery is currently the most effective surgical treatment for obesity since it is the only one that has demonstrated long-term loss of excess weight in a sustained manner, the re-mission of comorbidities, and an increase in hope and quality of life. The main surgical techniques in bariatric surgery currently performed are gastric bypass, a mixed technique consisting of a small gastric bag and an intestinal bypass, and vertical gastrectomy, a restrictive technique. Until now, there is no clear evidence regarding which surgical technique is the most appropriate for the majority of patients, since both bypass and gastrectomy offer similar results in terms of weight loss and resolution of comorbidities. Therefore, it will have to be an individualized decision in each case.

## Author Contributions

MJL and JF have been responsible for the conception of the work. PL has designed and planned the study, collecting data, analyzing it and interpreting it. MJL, JF and IC have been responsible for the writing of the article. SS has supervised the study as a whole and has been the guarantor that all the aspects that make up the manuscript have been reviewed and discussed among the authors, in order to be exposed with the maximum precision. All authors listed have made a substantial, direct, and intellectual contribution to the work and approved it for publication.

## Conflict of Interest

The authors declare that the research was conducted in the absence of any commercial or financial relationships that could be construed as a potential conflict of interest.

## Publisher’s Note

All claims expressed in this article are solely those of the authors and do not necessarily represent those of their affiliated organizations, or those of the publisher, the editors and the reviewers. Any product that may be evaluated in this article, or claim that may be made by its manufacturer, is not guaranteed or endorsed by the publisher.
